# Smart Sensorization Using Propositional Dynamic Logic

**DOI:** 10.3390/s22103899

**Published:** 2022-05-20

**Authors:** Salvador Merino, Alfredo Burrieza, Francisco Guzman, Javier Martinez

**Affiliations:** 1 Department of Applied Mathematics, University of Malaga, 29071 Malaga, Spain; jmartinezd@uma.es; 2 Department of Philosophy, University of Malaga, 29071 Malaga, Spain; burrieza@uma.es; 3 Department of Electrical Engineering, University of Malaga, 29071 Malaga, Spain; f_guzman@uma.es

**Keywords:** domotic, logic, qualitative reasoning, solar water heating, domestic hot water

## Abstract

The current high energy prices pose a serious challenge, especially in the domestic economy. In this respect, one of the main problems is obtaining domestic hot water. For this reason, this article develops a heating system applied to a conventional water tank in such a way as to minimize the necessary energy supply by converting it, under certain circumstances, into atmospheric. For this purpose, the domotic system has been equipped with sensors that automate the pressurization of the compartment and solenoid valves that regulate the external water supply. This design, to which different level sensors are applied, sends the information in real time to an artificial intelligence system, by means of deductive control, which recognizes the states of the system. This work shows the introduction of an extension of propositional dynamic logic in the field of energy efficiency. Thanks to this formalism, a qualitative control of the program variables is achieved by incorporating qualitative reasoning tools. On the other hand, it solves preventive maintenance systems through the early detection of faults in the installation. This research has led to the patenting of an intelligent domestic hot water system that considerably reduces energy consumption by setting disjointed heating intervals that, powered by renewable or non-renewable sources, are controlled by a propositional dynamic logic.

## 1. Introduction

In this paper we present an intelligent control system for the storage and supply of domestic hot water suitable for domotic installations. This system can take advantage of alternative energy sources (such as DHW solar panels and/or photovoltaic panels for heating the electrical resistance) in order to achieve the highest degree of sustainability and ecology. The behavior of this system can be represented by a logical formalism that allows to describe the processes of the system, to detect failures in it, and to facilitate the tasks of maintenance of the installation. The formalism used is very versatile and allows its application in very diverse areas of domotics, which we will demonstrate through some examples.

Current domotic control systems [1] and energy efficiency [2] have evolved from the simple activation and deactivation of loads [3] to having to consider combinations of states in sensors and actuators [4], determining the mode of operation of the same. An example of this can be found in the control of large photovoltaic solar farms [5], where possible reconfiguration [6] and maintenance [7] problems in the panels require precise measurement [8] and control of each unit [9] through separate activations or outages [10]. The profusion of states to be considered in some systems means that a process is needed to ensure that none of the circumstances that may occur in that process are ignored.

This is not something new; in fact, there are already languages that try it, such as GRAFCET, using logic gates [11]. This language is a procedure of synthesis and reduction of both the steps and the memory locations needed to automate an industrial process, using the existing digital simplification methods [12]. Its main problem is that if there is a malfunction of the deduced system, it is very difficult to determine where the error is, since the relationship between the resulting equation and the real logic of the system’s operation has been lost [13]. We need a formalism in which that does not happen, so that it can easily be determined where the dysfunction of the resulting equation is located. One of our fundamental aspirations in this paper is to introduce a logic that takes this aspect into account.

On the other hand, in domotic systems, many applications require incremental regulation in what are called “relative values” [14]. An example can serve to clarify these concepts: if we have an intelligent irrigation system, its operation will allow irrigation of it only when the humidity level of the soil requires it, and it can even take measurements in several locations to determine an average value of that humidity [15]. If the value of this humidity advises it, the irrigation system will be activated, but how long will it be irrigated? Normally, for a complete irrigation cycle to be carried out, it suffices that the activation threshold value has been reached, whether or not so much water is required. We need a reasoning system through which we can get it to irrigate, not according to a specific calendar and time, but in the fair value of humidity that our plants require [16]. For this reason, what we will undertake is to increase “a little” the necessary activation time until the desired humidity level is reached.

The examples presented prove the importance of considering qualitative aspects that require to have an intelligent control. The advantage of using qualitative information is that it allows us to handle situations in which we do not need precise numerical information of the system to make decisions [17]. In doing so, we incorporated tools of the qualitative reasoning (QR) to domotic, an approach with many applications in artificial intelligence (AI) (see, for example, [18]).

We propose in this paper a formal representation of domotic systems dealing with all these issues and applied to our invention. Our purpose is to overcome both errors and omissions that may have occurred during its application, in order to be able to contrast what was programmed with the intended rational operation. In addition, the formalism employed allows the incorporation of qualitative information on the status of certain variables and manages it to perform actions [19].

A domotic system can be modeled as a (finite) set of objects belonging to a building (windows, lights, taps, awnings, elevators, appliances, heating systems, etc.) or physical entities with measurable magnitudes (temperature of the water or of a room, the water flow of a tap, the intensity of light, etc.). We will express the properties of states by labels (state labels) that can be numerical (if we want to be precise) or qualitative. Qualitative state labels can be either dichotomous (e.g., open or closed) as well as with gradations (e.g., little, quite, many, …) [20].

The representation of the behavior of a domotic system can be performed by what are called *scenes*. A scene is an abstract representation of the behavior of a domotic system at a given time, taking into account the state of the variables that we use to describe it. Since a domotic system is a dynamic system, it undergoes modifications, which gives rise to a transition between scenes. In our approach, this transit can be expressed through operations of composition between qualitative labels as values of our variables. For this purpose we will use a qualitative arithmetic based on absolute orders of magnitude (AOM) [21], in which different qualitative sum operations will be defined. The orders of magnitude arise from making some kind of partition of the real line into equivalence classes, where the numbers are grouped under the same category or qualitative class (large, medium, small, …). Arithmetic operations between qualitative classes depend on how the boundaries of these intervals are defined. A study of the properties of different tables for the qualitative sum can be found in [22].

An appropriate formalism to attend transitions between states as well as qualitative arithmetic operations which can generate these states is propositional dynamic logic (PDL) [23]. An advantage of using PDL as a base is that we can make the transition between scenes using a program. PDL also allows us to describe the interaction between actions of computer domain and propositions about situations that we consider relevant for the system that we are dealing with. In addition, it is easy to integrate qualitative arithmetic into PDL through programs. The extended PDL with this feature will be called *Dom*.

Other logics are also designed to deal with state transitions (e.g., transaction logic [24], event calculus [25], situation calculus [26]); however, we do not need represent specifically the time (as event calculus or situation calculus). In addition, many of the aforementioned are predicate languages [27], which leads to problems with decidability. In our case we have a decidable propositional language, PDL [28], for which has been developed *model checking* techniques [29] and *automated theorem proving* techniques [30] through application examples [31].

The article is structured as follows: Section 2 introduces the intelligent multi-behavioral solar water heating system. Section 3 is dedicated to the logic *Dom*, created specifically to solve domotic systems. In Section 4, the formalization of the intelligent solar water heating system is carried out, using propositional dynamic logic. The last section is devoted to conclusions.

## 2. Design of an Intelligent Solar Water Heating System (ISWHS)

The system presented is a hot water accumulator model adapted for solar collector systems which also incorporates an electrical resistance to heat the water of the accumulator when it is required. In actuality, there are multiple systems of this type and in all cases the consumption of hot water is replaced by cold water entering from the external network causing a decrease in temperature, while the electrical resistance tries to compensate for this loss of heat. The problem is that it is not always possible to maintain a temperature acceptable to the user, and, in addition, there is a significant consumption of electrical energy.

Our system solves these problems, is innovative in its design and operation (there is a patent: [32]), minimizes the consumption of electrical energy, and ensures the maximum efficiency of solar panels. Briefly stated, we start from an initial situation in which the accumulator is completely full, the inlet valve of cold water from the network is open, and the air inlet/outlet valve and hot water outlet valve are closed. When there is no solar supply and there is hot water consumption, the system will allow, by means of a set of solenoid valves (the air inlet/outlet valve and the hot water outlet valve open, meanwhile the cold water closes), that the accumulator is emptied to a certain level, significantly lower than the maximum (*first stage*). At that moment, the *second stage* begins, during which cold water is allowed to enter from the network and an electrical resistance can be placed into operation. When the water reaches a predetermined intermediate level (*third stage*), the cold water valve closes. With the consumption, the water of the accumulator descends again to the low level (of the second stage) and the process is repeated, oscillating the water between the two levels (low and intermediate). This process continues as long as there is no caloric inlet from the solar panels. When the solar collector circuit supplies hot water again, the cold water circuit is opened and the electrical resistance (*fourth stage*) is deactivated. When the water reaches the maximum level (*fifth stage*), the system returns to the initial state (closing the air inlet/outlet valves and the hot water inlet valve).

**Figure 1:**
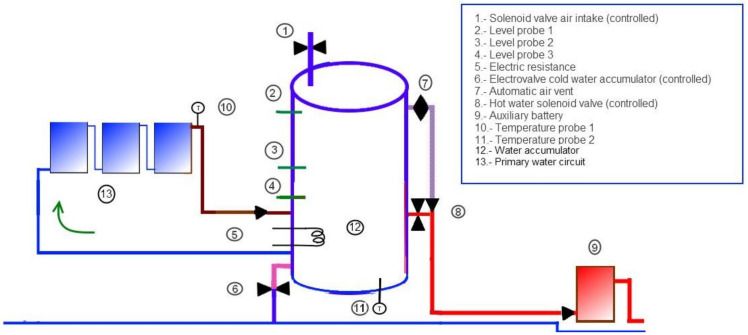
Components description.

We note that the fourth stage can begin in any of the first three stages above, depending on how long the solar collectors circuit is inactive. The second hot water outlet (⑦ in Figure 1) is opened and closed automatically and its behavior has not been described, as it lacks logical control.

This system has several advantages over traditional systems:  

- In traditional systems, the cold water that enters from the network is heated to replenish the hot water consumed when there is not enough solar radiation. In our case, the accumulator is left emptying and the only contribution of the electrical resistance is usually used to keep the water in the accumulator hot (but at a low level of the accumulator volume). Once the solar contribution is recovered, cold water is introduced from the network but it is heated by solar energy. This is a significant energy saving.
- As no cold water is introduced during the first stage, in which the water of the accumulator is emptied by the consumption of hot water by the users, there is no decrease of the water temperature in the accumulator by the inlet of cold water, which is normal since the cold water inlet valve is closed. This system keeps the water hot *for longer*.
- During the second stage, the hot water consumed is replaced by cold water from the network heated by the electrical resistance. However, since the water volume of the accumulator is lower (not the entire tank, but a quarter), the heat input of the resistance is also lower than a traditional system. Therefore, the energy saving also occurs at this stage.
- During the third stage, the accumulator is filled with cold water from the network to return to the initial situation and the solar collectors are heating the water. The temperature of the water inside the accumulator decreases and therefore the temperature difference in relation to the water temperature of the solar collectors is maximum. For this reason, this design ensures maximum efficiency of the solar panels, since the differential of temperature of the water passing through the panels with the water of the accumulator will always be maximum. This produces the maximum performance of the solar collectors.

### 2.1. System Description

The system presented (see Figure 1) aims to achieve maximum energy efficiency. Unlike traditional ones, where the accumulator has four inlets/outlets, the current one has six. The conventional inlets/outlets are those that connect the accumulator ⑫ to the solar collectors or primary circuit ⑬, a cold water inlet of the network controlled by the solenoid valve ⑥ and a hot water outlet controlled by the solenoid valve ⑦. The two additional inlets/outlets are one to allow air to enter the tank ① and another one for the hot water outlet ⑧. The solenoid valves or non-return valves have the purpose of controlling the opening/closing of the inlets/outlets. In total there are three solenoid valves, two of which we have already mentioned, and are as follows:  

- The air inlet to the accumulator (A).
- The cold water inlet from the network to the accumulator (CW).
- The hot water outlet from the accumulator (HW)
  (located in the figure in the places ①, ⑥, and ⑧, respectively).

We also have three water level probes inside the accumulator (numbers ②, ③, and ④ and an electrical resistance (ER) ⑤).

In this system we will consider three types of temperature:

- The temperature of the water inside the accumulator (AT), measured by the temperature probe ⑪.
- The temperature of the water passing through the panels (PT) measured by the temperature probe ⑩.
- The temperature requested by the user or *setpoint* temperature (ST).

### 2.2. System Operation

The general operation is based on the comparison between three different types of temperature and the levels reached by the water inside the accumulator, which will condition the opening and closing of three solenoid valves and the activation or deactivation of an electrical resistance.

We start with a situation where the accumulator is full of water and the CW is open while A and HW are closed.  

**Figure 2:**
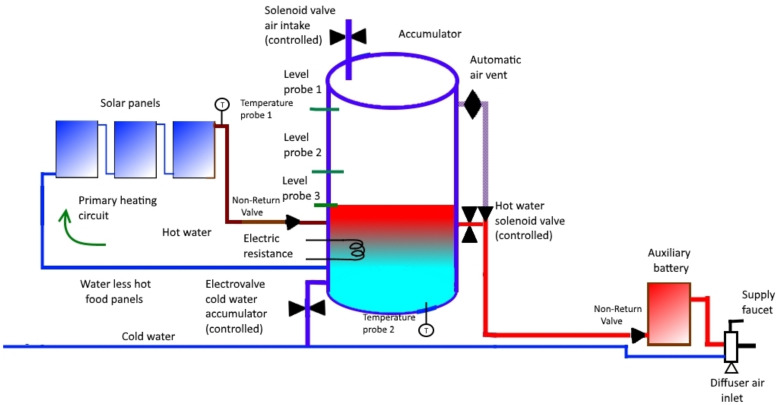
Functioning scheme 1.

**Figure 3:**
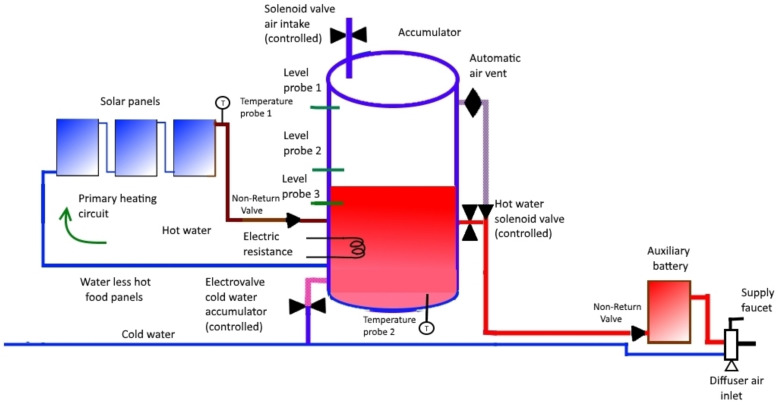
Functioning scheme 2.

**Figure 4:**
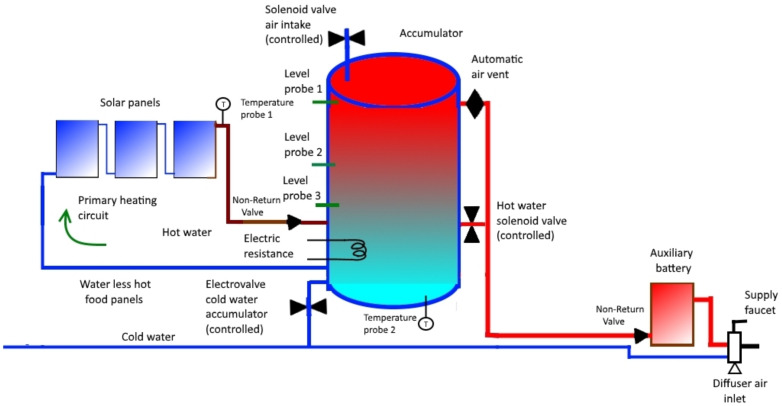
Functioning scheme 3.

1. The operation of the primary circuit is automatic: when the temperature of the water leaving the panels (the same water passes through them, one after another, since they are in series) is greater than that of the water inside the accumulator, water circulates due to the thermosyphon effect. When this is not the case, the water will not flow. This does not need control since it is a physical phenomenon.
2. Let us describe the operation of the rest of the circuit:
  - When AT is greater than PT, the three solenoid valves will be activated as follows: CW closes, while A and HW open.
    The three valves remain in their respective states until the water level inside the accumulator reaches the position of the level probe 3 (LP3) (see Figure 2).
    At that time, CW opens and we proceed to activate, or not, the electrical resistance (ER), according to the comparison between AT and ST as follows:
    - If AT is lower than ST, then ER is activated to the *hot* state;
    - If AT = ST, then ER is activated to the *normal* state;
    - if AT is greater than ST, then ER must be deactivated (*cold* state).
    In addition, cold water will continue to enter until the water level inside the accumulator reaches the level probe 2 (LP2). In this case, CW is closed.
    This process will allow the water level to be between probe 2 and 3 while the hot water is being used to consume through the lower outlet of the accumulator (see Figure 3).
  - When PT is greater than or equal to AT, CW will open and ER will be deactivated; when the water reaches level probe 1 (LP1), A and HW will close (see Figure 4).
  It should be noted that this process will lead to AT being greater than or equal to PT (again, previous case). Recall that previously we started from the situation we have now reached with respect to the three solenoid valves: CW is open while A and HW are closed; this describes a circuit.

With this system we can guarantee the maximum efficiency of the solar panels, since the temperature differential with the water inside the accumulator will always be maximum. In addition, the cooling of the water inside the accumulator is also prevented by the entry of cold water from the network, a situation that occurs when hot water is consumed in conventional installations.

The operation of the above described system can be expressed by the following Algorithm 1:
**Algorithm 1** Procedure of an intelligent water heating system (PIWHS)Initial situation: the accumulator is full of water, CW is open, and A and HW are closed.**begin**    //Compare AT and PT//**while** *true* **do**   **if** AT > PT **then**         **Close** CW and **Open** A and HW;         **if** the water level of the accumulator reaches the probe 3 **then**            Open **CW**;            **if**               AT < ST ⇒ pass ER intensity to *hot*;               AT = ST ⇒ pass ER intensity to *normal*;               AT > ST ⇒ **Turn off** ER;//ER must be in *cold* state//            **fi**         **end if**         **if** the water level of the accumulator reaches the probe 2 **then**            **Close** CW;         **end if**   **else**       //AT ≤ PT//         **Open** CW and **Turn off** ER;         **if** the water level of the accumulator reaches the probe 1 **then**               **Close** A and HW;         **end if**   **end if****end while****end**

## 3. The Logic *Dom*

When using tools for solving domotic problems, such as the one described above, the application of propositional dynamic logic will be used. In this case it will be called *Dom* logic.

### 3.1. Notation and Basic Concepts

We will use two types of expressions. On the one hand, expressions such as $\left(x_{1},\ldots ,x_{k}\right)$ are called *scene formulas*, where each $x_{i}$ is an element of a set of state labels $L_{i}$. These labels express gradations of the physical magnitudes (e.g., light intensity, tap water flow, room temperature, etc.) or certain states in which the element of the system are found (e.g., the elevator is on second floor, the tap is closed). For example, consider a domotic system in which we want to refer to the state (off or on) of the light and the degree of temperature of a room. We can consider a scene $x=(x_{1},x_{2})$, where $x_{1}$ is the state of light within the range $L_{1}=\left\{\mathrm{on},\mathrm{off}\right\}$ and $x_{2}$ is the state of the room temperature within the range $L_{2}=\left\{\mathrm{very}\;\mathrm{low},\mathrm{low},\mathrm{normal},\mathrm{high},\mathrm{very}\;\mathrm{high}\right\}$. Then the scene (on,high) means that the light is on and the room temperature is high. In actuality, in a scene formula we wish to represent only certain relevant aspects of the states in which a domotic system is found; however, many other things can happen that are beyond the description of the scene formula and which, eventually, we may be interested in taking into account. That expressed by the previous scene (on,high) could happen, for example, whether the room temperature is pleasant for the homeowner or not, as if one of the lamps does not work, or all are in perfect condition, etc.

In a scene formula we can use a special label, the *empty label*, denoted by “−”; its intuitive meaning is to represent that there is no information about the state of a particular component of the scene or that it is not necessary to specify that state (because it has not been modified from a previous specification or for any other reason). The label “−” also allows us to create a common scene to specific scenes, which implies a greater degree of abstraction. Thus, (on,−) refers as concrete instances to (on, very-low), (on, low), etc. Note that “−” (which is not actually a state label) in the latter sense comes to play the logical role of a variable.

Given a scene formula $x=(x_{1},\ldots ,x_{k})$, we will use the expression $x^{\left\{\ell_{1}/1,\ldots ,\ell_{k}/k\right\}}$ to indicate that we replace *simultaneously* in *x* each component $x_{i}$ for $\ell_{i}$ (we admit the possibility $x_{i}=\ell_{i}$. This indicates that there is no substitution in the *i* position. It will be useful to consider this possibility), which we denote abbreviated by $\ell_{i}/i$ and where $\ell_{i}$ denotes a state label in $L_{i}$. The expression $x^{\left\{p_{1},\ldots ,p_{r}\right\}}$ means that we highlight in *x* the positions $p_{1},\ldots ,p_{r}\in \left\{1,\ldots ,k\right\}$. Similarly, the expression $x^{\left\{\epsilon^{}1/p_{1},\ldots ,\epsilon^{}r/p_{r}\right\}}$, with highlighting positions $p_{1},\ldots ,p_{r}$, is obtained by replacing *simultaneously* the label of each position $p_{i}$ of *x* by $\epsilon^{}i$.

**Figure 5:**
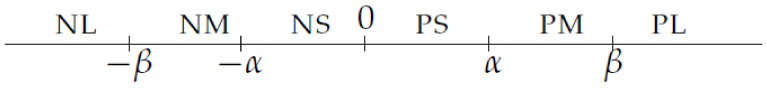
Partition of real line in qualitative classes.

The composite operations to make transitions among scenes are performed between the elements of each set of state labels $L_{i}$ with a special set of labels called *gradient labels*, denoted by $L_{G}$. We can use different sets of gradient labels for different set of state labels depending on the application in mind. As an example of set $L_{G}$ of qualitative labels, a classical partition of R in qualitative classes appears in Figure 5 (see [20,21]):

$$\begin{array}{ccc} \mathrm{NL}=(-\infty ,-\beta ) & & \mathrm{PS}=(0,\alpha ]\\ \mathrm{NM}=[-\beta ,-\alpha ) & 0=[0] & \mathrm{PM}=(\alpha ,\beta ]\\ \mathrm{NS}=[-\alpha ,0) & & \mathrm{PL}=(\beta ,+\infty ) \end{array}$$

The labels means “negative large” (nl), “negative medium” (nm), “negative small” (ns), “zero” (0), “positive small" (ps), “positive medium” (pm), and “positive large” (pl). The constants α,β are real numbers that are used to delimit the equivalence classes (the particular criteria to choose these numbers would depend on the application in mind).

Now, we introduce some notation. If $\ell_{1}$ and $\ell_{2}$ are two labels (of state or gradient), then $\ell_{1}\ll \ell_{2}$ means that $\ell_{1}$ is previous to $\ell_{2}$, that is, for any $x\in \ell_{1}$, and $y\in \ell_{2}$, we have that x<y. In the previous case, we have the order NL≪NM≪NS≪0≪PS≪PM≪PL. When we use state labels that express dichotomy such as on, off, we will consider off ≪ on. The notation ≫ indicates the inverse relationship of ≪; also, $\ell_{1}\underline{\;\ll \;}\ell_{2}$ means that $\ell_{1}\ll \ell_{2}$ or $\ell_{1}=\ell_{2}$.

A set of gradient labels $L_{G}$ is intended to increase or decrease the magnitudes of the variables of a scene operating on the status labels using composition. Thus, using the previous partition as set of gradient labels, if the light has a *low* intensity (state label), we can increase it a *little*, that is, with a small number or belonging to ps (gradient label), which may result that the light remains within the range of *low* (even if it has increased in intensity) or pass to the *normal* state (new label status). In order to perform the compositing operations in this case, we will translate the state labels into gradient labels and use a previously defined qualitative sum operation according to our interests.

There are several types of expressions that we can form when performing operations of composition in the positions of a scene formula. Therefore, the notation $p_{i}+\ell_{i}^{+}:p_{i}$ indicates that in the position $p_{i}$ we must perform a composition operation (sum) between the state label $\ell_{p_{i}}\in L_{p_{i}}$ and the (positive) label $\ell_{i}^{+}$. The nature of + (numerical or qualitative) obviously depends on the labels involved. The sum is qualitative if at least one of the factors is qualitative. If both labels are qualitative, then $\ell_{i}^{+}$ is always a (positive) *gradient* label and the operation must always generate a qualitative state label $\ell_{p_{i}}^{\prime }\in L_{p_{i}}$ such that $\ell_{p_{i}}\;\underline{\ll \;}\;\ell_{p_{i}}^{\prime }$. If $\ell_{p_{i}}^{\prime }\in L_{p_{i}}$ is qualitative and $\ell_{i}^{+}$ is numerical, then the result of the composition is also a qualitative state label $\ell_{p_{i}}^{\prime }\in L_{p_{i}}$ such that $\ell_{p_{i}}\;\underline{\ll \;}\;\ell_{p_{i}}^{\prime }$. In the same sense, it occurs if $\ell_{p_{i}}^{\prime }\in L_{p_{i}}$ is numerical and $\ell_{i}^{+}$ is qualitative.

Similarly, $p_{i}+\ell_{i}^{-}:p_{i}$ indicates that in the position $p_{i}$, we compose its corresponding state label $\ell_{p_{i}}\in L_{p_{i}}$ with the (negative) gradient label $\ell_{i}^{-}$, which generates a state label $\ell_{p_{i}}^{\prime }\in L_{p_{i}}$ such that $\ell_{p_{i}}^{\prime }\;\underline{\ll \;}\;\ell_{p_{i}}$.

### 3.2. Syntax

The language of *Dom* is an extension of the language of PDL with a new set of atomic formulas and specific atomic programs. Concretely, *Dom* contains the propositional operators → (material implication) and ⊥ (falsity), the program operators ; (composition), ∪ (choice), and ${}^{*}$ (iteration) and the mixed operators [] (necessity) and ? (test). As defined symbols we have ⊤ (truth), ∧ (and), ∨ (or), ↔ (if and only if), and 〈〉 (possibility) as usual. Formulas and programs are built inductively, using the previous operators, from P (a set of alphanumeric chains), $L=L_{1}\times \cdots \times L_{k}$ (a set of scene formulas) being each $L_{i}(1\leq i\leq k$) a finite set of state labels and a specific parametrized program (called *regulator*) consisting of several subroutines that analyze below (although *regulator* can be declared in subprograms we consider it an atomic program, since it requires the simultaneity of all the subprograms that form it, so that it can not be obtained from these with the classic PDL operations as ; or ∪).

If $x=(x_{1},\ldots ,x_{k})$ is a scene formula, then we have as programs the following ones:

- ${\mathrm{Inc}}_{x}^{\left\{p_{1}+\ell_{1}^{+}:p_{1},\ldots ,p_{n}+\ell_{n}^{+}:p_{n}\right\}}$ (*increase*): This program means that we must increase the level of the values of the positions $p_{1},\ldots ,p_{n}$ of *x*, respectively, by the positive labels $\ell_{1}^{+},\ldots ,\ell_{n}^{+}$, using a sum operation + (qualitative or numerical). A simultaneous replacement of $p_{i}$ by $p_{i}+\ell_{i}^{+}$ is carried out; e.g., increase a *little* the temperature of the living room.
- ${\mathrm{Dec}}_{x}^{\left\{p_{1}+\ell_{1}^{-}:p_{1},\ldots ,p_{n}+\ell_{n}^{-}:p_{n}\right\}}$ (*decrease*): This program means that we must decrease the level of the values of the positions $p_{1},\ldots ,p_{n}$ of *x*, respectively, by the negative labels $\ell_{1}^{-},\ldots ,\ell_{n}^{-}$, using a sum operation + (qualitative or numerical); e.g., decrease *much* the intensity of the kitchen light.
- ${\mathrm{Change}}_{x}^{\left\{\ell_{p_{1}^{\prime }}/p_{1},\ldots ,\ell_{p_{n}^{\prime }}/p_{n}\right\}}$ (*change*): This program means that we must replace *simultaneously* the state labels of the positions $p_{1}$…$p_{n}$ of *x* (i.e., $\ell_{p_{1}}$, …, $\ell_{p_{n}}$), respectively, by the state labels $\ell_{p_{1}^{\prime }}$, …, $\ell_{p_{n}^{\prime }}$ (e.g., set the room temperature to $20{\;}^{\circ }$C, …).
- ${\mathrm{Watch}}_{x}^{\left\{p_{1},\ldots ,p_{n}\right\}}$ (*watch*): This program means that we simply *watch* the state labels of the positions $p_{1},\ldots ,p_{n}$ of *x*; it is assumed that these would be maintained if there are no actions that change them. The purpose of this program is to explicitly check that these labels do not change even if those in other positions do.

The following program collects all of the above in a single order and is defined:

- ${\mathrm{Reg}}_{x}^{\Psi_{1},\Psi_{2},\Psi_{3},\Psi_{4}}$ (“*regulator*”), where $\Psi_{1}=\left\{p_{1}+\ell_{1}^{+}:p_{1},\ldots ,p_{l}+\ell_{l}^{+}:p_{l}\right\}$
  $\Psi_{2}=\left\{q_{1}+\ell_{1}^{-}:q_{1},\ldots ,q_{m}+\ell_{m}^{-}:q_{m}\right\}$
  $\Psi_{3}=\left\{\ell_{p_{1}^{\prime }}^{•}/p_{1}^{\prime },\ldots ,\ell_{p_{n}^{\prime }}^{•}/p_{n}^{\prime }\right\}$
  $\Psi_{4}=\left\{q_{1}^{\prime },\ldots ,q_{o}^{\prime }\right\}$
  and $p_{1},\ldots ,p_{l},q_{1},\ldots ,q_{m},p_{1}^{\prime },\ldots ,p_{n}^{\prime },q_{1}^{\prime },\ldots ,q_{o}^{\prime }$ are positions of *x*, all different, and at least one $\Psi_{i}$ (1≤i≤4) is not empty.

That is, *regulator* executes the actions of ${\mathrm{Inc}}_{x}^{\Psi_{1}},{\mathrm{Dec}}_{x}^{\Psi_{2}},{\mathrm{Change}}_{x}^{\Psi_{3}}$, and ${\mathrm{Watch}}_{x}^{\Psi_{4}}$ simultaneously, something very important in domotic, and without problems, because the positions of the different subprograms that include it are all different. This allows to create a final scene with all the actions executed of these programs without going through intermediate scenes, which would result in case of proceed sequentially.

Note that we can define the previous programs in terms of *regulator* by a very simple procedure: place the empty set of operations with labels in the corresponding part. Thus, we define

$$\begin{array}{cc} {\mathrm{Inc}}_{x}^{\Psi_{1}}{=}_{def}{\mathrm{Reg}}_{x}^{\Psi_{1},⌀,⌀,⌀}\; & {\mathrm{Dec}}_{x}^{\Psi_{2}}{=}_{def}{\mathrm{Reg}}_{x}^{⌀,\Psi_{2},⌀,⌀}\\ {\mathrm{Change}}_{x}^{\Psi_{3}}{=}_{def}{\mathrm{Reg}}_{x}^{⌀,⌀,\Psi_{3},⌀}\; & {\mathrm{Watch}}_{x}^{\Psi_{4}}{=}_{def}{\mathrm{Reg}}_{x}^{⌀,⌀,⌀,\Psi_{4}} \end{array}$$

In general, we will use an expression such as ${\mathrm{Inc}}_{x}^{\Psi }$, ${\mathrm{Dec}}_{x}^{\Psi }$, etc., when we want to refer to one of these programs without specifying the set of substitutions that the program performs in the *x* scene. With this in mind, we can introduce new specific programs from ${\mathrm{Change}}_{x}^{\Psi }$ to treat dichotomous states, so we have

- ${\mathrm{On}}_{x}^{\left\{p_{1},\ldots ,p_{n}\right\}}$ (“turn on all the positions $p_{1},\ldots ,p_{n}$ of *x*”).
- ${\mathrm{Off}}_{x}^{\left\{p_{1},\ldots ,p_{n}\right\}}$ (“turn off all the positions $p_{1},\ldots ,p_{n}$ of *x*”).

That is, we have

${\mathrm{On}}_{x}^{\left\{p_{1},\ldots ,p_{n}\right\}}{=}_{def}{\mathrm{Change}}_{x}^{\left\{\mathrm{on}/p_{1},\ldots ,\mathrm{on}/p_{n}\right\}}$

and

${\mathrm{Off}}_{x}^{\left\{p_{1},\ldots ,p_{n}\right\}}{=}_{def}{\mathrm{Change}}_{x}^{\left\{\mathrm{off}/p_{1},\ldots ,\mathrm{off}/p_{n}\right\}}$. 

We can also define *mixed dichotomous programs* that combine the actions of the previous ones. For example,

- ${\mathrm{Onoff}}_{x}^{\Psi_{1},\Psi_{2}}$. This program combines ${\mathrm{On}}_{x}^{\Psi_{1}}$ and ${\mathrm{Off}}_{x}^{\Psi_{2}}$, where $\Psi_{1}$ and $\Psi_{2}$ are sets of distinguished positions of different *x*.
- ${\mathrm{Onoff}}_{x}$ (“exchange on and off”) is a generic modification of the previous program ${\mathrm{Onoff}}_{x}^{\Psi_{1},\Psi_{2}}$, that simply does not require previous identification of the positions to be activated/deactivated (the system reads the status of all positions and performs this order automatically, going through all of them). In actuality, $\Psi_{1}$ are all positions in the scene with off and $\Psi_{2}$ are all positions in the scene with on. This program *activates* in *x* all positions that are deactivated and at the same time *deactivates* all positions that are enabled; e.g., one result would be to turn all lights on (that are off) and turn off all lights (that are on) simultaneously.

Now we give some definitions about scene formulas.

**Definition** **1.** *Let $x=(x_{1},\ldots ,x_{k})$ and $y=(y_{1},\ldots ,y_{k})$ be scene formulas. We say that x is different from y if $x_{i}\neq y_{i}$ for some i∈{1,…,k}.*

**Definition** **2.** *Let $x=(x_{1},\ldots ,x_{k})$ and $y=(y_{1},\ldots ,y_{k})$ be scene formulas and let us consider the program ${\mathrm{Reg}}_{x}^{\Psi_{1},\Psi_{2},\Psi_{3},\Psi_{4}}$. We say that y is x-regulated with respect to (abbreviated wrt) $\Psi_{1}$–$\Psi_{4}$ if for every i with (1≤i≤k) we have one of the following cases:*

- *$y_{i}=x_{i}+\ell_{i}^{+}$, where $x_{i}+\ell_{i}^{+}\in \Psi_{1}$, + is an operation defined for $L_{i}$ and $\ell_{i}^{+}$ is a positive label of the set $L_{G}$ (chosen for $L_{i}$).*
- *$y_{i}=x_{i}+\ell_{i}^{-}$, where $x_{i}+\ell_{i}^{-}\in \Psi_{2}$, + is an operation defined for $L_{i}$ and $\ell_{i}^{-}$ is a negative label of the set $L_{G}$ (chosen for $L_{i}$).*
- *$y_{i}=x_{i}^{\left[l_{i}/x_{i}\right]}$, where $l_{i}/x_{i}\in \Psi_{3}$ and $l_{i}\in L_{i}$.*
- *$y_{i}=x_{i}$ and $x_{i}\in \Psi_{4}$.*

**Example** **1.** *Suppose a domotic system is composed of the open/closed properties of a window and its degree of openness. For this, we use two sets of labels: $L_{1}=\left\{\mathrm{on},\mathrm{off}\right\}\;(\mathrm{open}/\mathrm{close}\;\mathrm{states})$ and $L_{2}=\left\{\mathrm{very}-\mathrm{little},\mathrm{little},\mathrm{normal},\mathrm{much},\mathrm{very}-\mathrm{much}\right\}$. As a set of gradient labels $L_{G}$ for $L_{2}$ we have*

{NL,NM,NS,0,PS,PM,PL}

*The normal label denotes a previously established standard minimum aperture. The language contains state formulas such as $\left(x_{1},x_{2}\right)$, where $x_{1}$ refers to the open/closed state of the window and $x_{2}$ to the degree of opening of the window. Then the formula*

$$[\left(\mathrm{off}\;,-\right)?;{\mathrm{Change}}_{\left(\mathrm{off},-\right)}^{\left\{\mathrm{on}/1,\mathrm{normal}/2\right\}}]\left(\mathrm{on},\mathrm{normal}\right)$$

*indicates that any action consisting of opening the window (initially closed) results in a state in which the window is open with a normal (stipulated or predetermined) degree of aperture. Then, the formula*

$$\left[{\mathrm{Inc}}_{\left(\mathrm{on},\mathrm{normal}\right)}^{\left\{\mathrm{normal}+ps:2\right\}}\right]\left(\mathrm{on},\mathrm{normal}\right)\vee \left(\mathrm{on},\mathrm{much}\right)$$

*indicates that any action that opens the window a little more will lead to it either remaining within the normal parameter of opening or change to a new state, i.e., to become quite open (this requires us to predefine a matrix where a + operation is handled between the state labels and gradient labels so that normal+PS leads tonormalormuch). The notationnormal+*ps*:2 indicates that in the second position of the scene (on,normal) its label is replaced by some label resulting from the compositionnormal+*ps.

*Now, we will express a more complex situation. Let us consider the atoms p,q with the respective meanings “the heating is on" and “it is economically beneficial". The following program (denoted by a) expresses the action of closing the window, and also, while the heating is on, makes sure the window is closed:*

$$\left(\mathrm{on},-\right)?;{\mathrm{Off}}_{\left(\mathrm{on},-\right)}^{\left\{1\right\}};{\left(p?;{\mathrm{Watch}}_{\left(\mathrm{off},-\right)}^{\left\{1\right\}}\right)}^{*};\neg p?$$

*Note that $\left(\mathrm{on},-\right)?;{\mathrm{Off}}_{\left(\mathrm{on},-\right)}^{\left\{1\right\}}$ indicates that if the window is open then it closes, and*

$${\left(p?;{\mathrm{Watch}}_{\left(\mathrm{off},-\right)}^{\left\{1\right\}}\right)}^{*};\neg p?$$

*is an instruction of type <<while>>. Then the formula [a]q means that the result of the previous action is always something economically beneficial.*

### 3.3. Semantics

A *model* M is a tuple (W,m) where *W* is a nonempty set of states (called, more specifically, *domotic states*). We will use letters as u,v,w (with or without indexes) as representing states. By convenience, each element u∈W is to be understood as a domotic state labeled by elements of *L*.

1. The meaning function *m* is required to fulfill the following:
  (a) m(p)⊆W, for every atomic formula p∈P.
  (b) m(x)⊆W, for every scene formula x∈L and m(x)≠⌀.
  (c) For all w∈W there exists a scene formula x∈L such that w∈m(x) (all the states of a model are described by a scene formula).
  (d) For all w∈W and x∈L, if w∈m(x), then *x* is unique, that is, for all y∈L different from *x* (in the sense of Definition 1) we have w∉m(y).
2. We define now the semantics of the specific program *regulator*. if $x=(x_{1},\ldots ,x_{k})$ is a state formula, then
  -
$m\left({\mathrm{Reg}}_{x}^{\Psi_{1},\Psi_{2},\Psi_{3},\Psi_{4}}\right)\left(m\left(x\right)\right)\subseteq$

    $\{v|v\in m\left(y\right)\;\mathrm{and}\;y\;\mathrm{is}\;x-\mathrm{regulated}\;\mathrm{wrt}\;\Psi_{1}$-$\Psi_{4}\}$
3. Finally, if φ and ψ are formulas and a,b are programs, then we have the following:
  (a) m(φ→ψ)=(W\m(φ))∪m(ψ);
  (b) m(⊥)=⌀;
  (c) m([a]φ)={w∈W∣forallv∈W,if(w,v)∈m(a)thenv∈m(φ)};
  (d) m(a∪b)=m(a)∪m(b);
  (e) m(a;b)=m(a)∘m(b) = {(w,v)∣thereexistsu∈Wsuchthat(w,u)∈m(a)and(u,v)∈m(b)};
  (f) $m\left(a^{*}\right)=m{\left(a\right)}^{*}$ (reflexive and transitive closure of relation m(a));
  (g) m(φ?)={(w,w)∣w∈m(φ)}.

The semantic concepts of *satisfiability*, *valid in a model*, and *valid* are the usual ones.

## 4. Formalizing ISWHS

In this section we will express the operation of the system using the programming language of the logic.

### 4.1. System States

We will use scene formulas with seven state variables $\left(x_{1},x_{2},x_{3},x_{4},x_{5},x_{6},x_{7}\right)$, where

- $x_{1}$ denotes the temperature of the water accumulated in the accumulator (AT).
- $x_{2}$ denotes the temperature of the water passing through the solar panels (PT).
- $x_{3}$ denotes the status of the cold water solenoid valve of the network to the accumulator (CW).
- $x_{4}$ denotes the status of the solenoid valve in the upper part of the accumulator (A).
- $x_{5}$ denotes the status of the solenoid valve of the lower hot water outlet of the accumulator (HW).
- $x_{6}$ denotes the status of the electrical resistance (ER).
- $x_{7}$ denotes the set point temperature defined by the user (ST).

We will also use “propositional constants” to denote a certain behavior of the system:

- si denotes that the water level inside the accumulator reaches the probe of the upper level *i* (i=1,2,3).

The sets of state labels $L_{1},\ldots ,L_{7}$ are as follows:

- $L_{1}=L_{2}=\left\{\mathrm{vc},c,n,h,\mathrm{vh}\right\}$.
- $L_{3}=L_{4}=L_{5}=\{\mathrm{on}$, off}.
- $L_{6}=\left\{c,n,h\right\}$.
- $L_{7}$ = a finite subset of N (to choose).

As gradient labels we have the ones established in the partition of the real line made in Section 3.1, namely, {NL,NM,NS,0,PS,PM,PL}. The intuitive meanings of the state labels are vc=very-cold; c=cold; n=normal; h=hot; vh=very-hot. We have the following label orders. For $L_{i}\left(i=1,2\right)$: vc≪c≪n≪h≪vh; for $L_{j}\left(j=3,4,5\right)$: off≪on; and for $L_{6}$: c≪n≪h.

### 4.2. Composition Operations

In this section we introduce the composition operations. We start with the table for the gradient labels ($L_{G}$). Recall the partition made in Figure 5, where we consider NL=(−∞,−β), PS=(0,α], NM=[−β,−α), 0=[0] and PM=(α,β]. The $L_{G}$ partition can contain a different numerical set appropriate for each application (a subset of R). On the other hand, from now on, we will consider that β=2α.

In Table 1, the operation ${+}_{G}$ between qualitative classes yields either a qualitative class or a range of qualitative classes [X,Y] (where X≪Y), which we express, indicating all the qualitative classes that compose this interval separated by commas for clarity. For example, NS ${+}_{G}$PS provide us, as a result, the interval [NS, PS], comprising NS, 0, PS. On the other hand, we used 1 to denote the interval [NL, PL].

The operation ${+}_{G}$ verifies the following: ${+}_{G}$ is *consistent* with the usual real sum +, that is, for all qualitative classes, X,Y, and all x∈X,y∈Y, $X{+}_{G}Y$ corresponds to the smallest interval (in the sense of inclusion) formed by qualitative classes containing x+y (see [19,22]). For example, nm${+}_{G}$PM = [NS, PS].

In Table 1, Table 2 and Table 3, the state labels are translated to gradient labels to make the composition that we make in Table 1. In Table 2 we take vc = nl, c = nm, n = [ns, ps], h = pm, and vh = pl. In Table 3 we take c=[NL,NM], n=[NS,PS], and h=[PM,PL]. Once the composition is completed, we translate the result to state labels by choosing the smallest possible interval of state labels. For example, the composition $n{+}_{2}\mathrm{PS}$ in Table 3 is, in fact, $\left[\mathrm{NS},\mathrm{PS}\right]{+}_{G}\mathrm{PS}=\left[\mathrm{NS},\mathrm{PM}\right]$. This result comes from composing, using ${+}_{G}$, in each of the classes of the interval [ns, ps] with PS. Now, since the minimum interval in which [ns, pm] is included in terms of the classes in Table 3 is [n, h] = [ns, pl], we finally have this output.

Table 2 describes the behavior of both the water temperature of the solar panels and the water inside the accumulator. The composition Table 3 with the labels of $L_{6}$ (Table 2) allows us to calculate both the increase and the gradual decrease of the ER intensity. For instance, we took a scale for $L_{G}$ where α=5 and β=10. Now we consider the following projection of $L_{G}$ over $L_{1}\left(L_{2}\right)$ in Table 2: −β corresponds to 20 ${}^{\circ }$C, −α with 25 ${}^{\circ }$C, 0 with 30 ${}^{\circ }$C, α with 35 ${}^{\circ }$C and β with 35 ${}^{\circ }$C. Therefore, we have for $L_{G}$ the following partition nl = […, −10), nm = [−10, −5), ns = [−5, 0), [0] = 0, ps = (0, 5], pm = (5, 10], pl= (10, …] and for $L_{1}\left(L_{2}\right)$ the corresponding partition very cold = […, 20), cold = [20, 25), normal = [25, 35], hot = (35, 40], very hot = (40, …]. Now, adding, for example, a small number (say 3) to a normal temperature (n) can lead us to stay within that temperature or go to hot (h), depending on how close the system is to the limit 35.

### 4.3. Logical Description of System Behaviour

The *Dom* logic can describe the behavior of the system. For instance, let us assume a state where AT > PT, AT < ST, the valves CW, A, and HW are open, and ER has a normal intensity and water level reaches probe 3 (a state perfectly coherent with a correct run of the algorithm for the system). Thus, s3 and the scene x=(−,−,on,on,on,n,−) hold at that state. The procedure establishes then that a new state where the scene y=(−,−,on,on,on,h,−) holds must be reached. If we operate gradually, increasing the intensity a *little* (a small number) until reaching the hot level, the formula

$$\left(x\wedge s3\right)\to [{\left(\neg y?;{\mathrm{Inc}}_{x}^{\left\{6+ps:6\right\}}\right)}^{*};y?]\;y$$

describes this process.

### 4.4. Detecting Failures in System Operation

In this section we show how our formalism captures what *should not happen* if we want the system described to work correctly.

We will give some examples of significant failures of the system. We will consider transitions from a state *w* to a state *v*, placing below each state some formulas true at it. The failure detected is a trouble with a valve or the resistance. In the following we add new atoms of the type sij, with the intuitive meaning “the water inside the accumulator is between the level probes *i* and *j*”. Now, we list some troubles emphasizing in the scenes only the information more relevant.

The first case in the previous Table 4 states that in the scene x=(−,−,−,off,−,−,−), the component $x_{2}$ belongs to a qualitative class lower than the component $x_{1}$. At the state *w*, the formulas *x* and s1 are true. At the state *v*, the same scenes *x* and s12 are true. The program establishes that A should be opened if AT > PT (i.e., $x_{2}\ll x_{1}$). This means that the scene *x* must vary the component $x_{4}$ to on. However, at state *v*, valve A stays off. After a sufficient lapse of time the water will be between probes 1 and 2, so the situation at *v* should be a different scene of *x*, namely, (−,−,−,on,−,−,−). This means that, according to the behavior of the program at this stage, the formula $\langle \;{\mathrm{Onoff}}_{x}^{\left\{4,5\},\{3\right\}}\;\rangle \left(-,-,-,\mathrm{off},-,-,-\right)$ must be false at *w*. Therefore, if the procedure for the system is correct, we can suspect a failure in valve A. The rest of the cases are interpreted in a similar way. In general, for these troubleshooting tasks in a graph, model-checking techniques can be applied.

## 5. Conclusions

The main conclusions of this work are as follows:

**Efficiency**: The proposed solution increases the efficiency of domestic hot water heating systems using renewable energies. Normally, the thermal profitability of this type of installation, powered by solar energy, represents a saving of around 80% of that required for heating [1]. The use of the system presented in this paper represents an additional saving of (100−x)% (depending on the position *x* of the lowest level sensor with respect to the bottom of the tank).
**Multiplicity**: The water heater itself has three different behaviors, depending on its load level. In principle, when the solar thermal input is sufficient, it works as a conventional domestic hot water storage tank powered by solar thermal energy. In a second state, when the heat transfer fluid coming from the panels has a lower temperature than the water in the tank to be heated, it cuts off the external supply and becomes atmospheric, allowing the interior water to be used without any possible mixing with the water at a lower temperature from the network. In the third state, when a minimum level is reached, it will behave similar to an electric heater, powered by renewable or non-renewable energies, working on a minimum level of water compared to the original.
**Economy**: The system would allow an estimated global saving of $0.80\times \frac{100-x}{100}$ based on the conventional energy used for the heating process. In addition, it allows to use all the water contained in the tank at the maximum temperature reached (until reaching the minimum load level marked by the probe located in the lowest zone) without mixing it with the water coming from the supply network (which would cool the mixture at a considerable speed). Likewise, when the outside temperature does not allow the stored water to be heated, it considerably reduces the consumption of electrical energy by significantly reducing the volume of water that will be used from that moment onwards (it practically becomes a heating as the water flow passes through).
**Complexity**: The resolution by means of propositional dynamic logic has overcome the disadvantages of traditional systems to detect faults in the logical operation of the system. In addition, it contains programs that allow qualitative control of the states of the variables, with built-in qualitative addition operations, which gives the system great flexibility. This type of operation is very useful in home automation as many applications require qualitative regulation.

## 6. Patents

In parallel to this paper was developed the patent number ES2627209B2 [32]. 

## Figures and Tables

**Table 1:** Composition of components of $L_{G}$.

${+}_{G}$	NL	NM	NS	0	PS	PM	PL
nl	nl	nl	nl	nl	nl, nm	nl, nm, ns	1
nm		nl	nl, nm	nm	nm, ns	ns, 0, ps	ps, pm, pl
ns			nm, ns	ns	ns, 0, ps	ps, pm	pm, pl
0				0	ps	pm	pl
ps					ps, pm	pm, pl	pl
pm						pl	pl
pl							pl

**Table 2:** Composition of components of $L_{1}$ ($L_{2}$).

${+}_{1}$	NL	NM	NS	0	PS	PM	PL
vc	vc	vc	vc	vc	vc, c	vc, c, n	1
c	vc	vc	vc, c	c	c, n	n	n, h, vh
n	vc, c	vc, c, n	c, n	n	n, h	n, h, vh	h, vh
h	vc, c, n	n	n, h	h	h, vh	vh	vh
vh	1	n, h, vh	h, vh	vh	vh	vh	vh

**Table 3:** Composition of components of $L_{6}$.

${+}_{2}$	NL	NM	NS	0	PS	PM	PL
c	c	c	c	c	c, n	c, n	1
n	c	c, n	c, n	n	n, h	n, h	h
h	1	n, h	n, h	h	h	h	h

**Table 4:** Scenes and States.

Scene Formula *x*	*w*	*v*	Trouble
$x_{4}=\mathrm{off}$, $x_{2}\ll x_{1}$	x,s1	x,s12	A
$x_{5}=\mathrm{off}$, $x_{2}\ll x_{1}$	x,s1	x,s12	HW
$x_{3}=\mathrm{on}$, $x_{2}\ll x_{1}$	x,s2	x,s12	CW
$x_{2}\ll x_{1},x_{7}\ll x_{1},x_{6}=n$	x,s3	y,s2 (where $y_{6}\neq c$)	ER
